# USPIO-enhanced MRI of pelvic lymph nodes at 7-T: preliminary experience

**DOI:** 10.1007/s00330-019-06277-7

**Published:** 2019-06-14

**Authors:** Bart W. J. Philips, Rutger C. H. Stijns, Stefan H. G. Rietsch, Sascha Brunheim, Jelle O. Barentsz, Ansje S. Fortuin, Harald H. Quick, Stephan Orzada, Marnix C. Maas, Tom W. J. Scheenen

**Affiliations:** 1 Department of Radiology and Nuclear Medicine (766), Radboud University Medical Center, P.O. Box 9101, Nijmegen, The Netherlands; 2 Erwin L Hahn Institute for Magnetic Resonance Imaging, University Duisburg-Essen, 45141 Essen, Germany; 3 High-Field and Hybrid MR Imaging, University Hospital Essen, 45147 Essen, Germany; 4 Department of Radiology, Ziekenhuis Gelderse Vallei, Ede, The Netherlands

**Keywords:** Ferumoxtran-10, Lymph nodes, Pelvis, Metastasis, Magnetic resonance imaging

## Abstract

**Purpose:**

To evaluate the technical feasibility of high-resolution USPIO-enhanced magnetic resonance imaging of pelvic lymph nodes (LNs) at ultrahigh magnetic field strength.

**Materials and methods:**

The ethics review board approved this study and written informed consent was obtained from all patients. Three patients with rectal cancer and three selected patients with (recurrent) prostate cancer were examined at 7-T 24–36 h after intravenous ferumoxtran-10 administration; rectal cancer patients also received a 3-T MRI. Pelvic LN imaging was performed using the TIAMO technique in combination with water-selective multi-GRE imaging and lipid-selective GRE imaging with a spatial resolution of 0.66 × 0.66 × 0.66mm^3^. T_2_^*^-weighted images of the water-selective imaging were computed from the multi-GRE images at TE = 0, 8, and 14 ms and used for the assessment of USPIO uptake.

**Results:**

High-resolution 7-T MR gradient-echo imaging was obtained robustly in all patients without suffering from RF-related signal voids. USPIO signal decay in LNs was visualized using computed TE imaging at TE = 8 ms and an R_2_^*^ map derived from water-selective imaging. Anatomically, LNs were identified on a combined reading of computed TE = 0 ms images from water-selective scans and images from lipid-selective scans. A range of 3–48 LNs without USPIO signal decay was found per patient. These LNs showed high signal intensity on computed TE = 8 and 14 ms imaging and low R_2_^*^ (corresponding to high T_2_^*^) values on the R_2_^*^ map.

**Conclusion:**

USPIO-enhanced MRI of the pelvis at 7-T is technically feasible and offers opportunities for detecting USPIO uptake in normal-sized LNs, due to its high intrinsic signal-to-noise ratio and spatial resolution.

**Key Points:**

*• USPIO-enhanced MRI at 7-T can indicate USPIO uptake in lymph nodes based on computed TE images.*

*• Our method promises a high spatial resolution for pelvic lymph node imaging.*

## Introduction

Lymph nodes often are the first organs to which cancer metastasizes. It is important to detect metastatic nodes at an early stage as a signal of systematic disease, such that adequate treatment can be provided. Prostate cancer, rectal cancer, and gynecological cancers, the major cancers that occur within the pelvis, all metastasize primarily to pelvic lymph nodes [1] and their lymph node status in patients is still primarily diagnosed using invasive surgical techniques, such as a pelvic lymph node dissection [2].

Given its high sensitivity (82–96%) and specificity (93–98%) [3–7], MRI in combination with a lymphotropic ultrasmall superparamagnetic particles of iron oxide (USPIO) contrast agent may be a valid non-invasive alternative for pelvic lymph node staging and has recently gained renewed interest [8, 9]. In short, intravenously administered USPIOs accumulate in healthy lymph nodes, strongly attenuating the MR signal in T_2_^*^-weighted imaging. Suspicious lymph nodes without USPIO uptake retain their MR signal. Although the reported sensitivities and specificities of this technique are promising, they depend on study design, inclusion criteria, and reader experience [5, 10, 11]. Furthermore, the sensitivity decreases to 41% for lymph nodes smaller than 5 mm [4]. These studies were performed on 1.5-T MR systems in the early 2000s [4]. Later work [11, 12] already showed improved image quality at 3-T versus 1.5-T, indicating that higher field strengths can improve the detection of lymph node metastases, particularly for lymph nodes smaller than 5 mm [13–15]. Moreover, a higher spatial resolution may allow a better identification of lymph node resembling structures such as nerve ganglia.

New technologies such as 7-T MRI systems offer a substantial increase in intrinsic signal-to-noise ratio (SNR) [16]. Moreover, the decrease of T_2_^*^ in non-metastatic lymph nodes due to USPIO uptake is more pronounced at higher field strengths due to increased susceptibility effects [17]. These effects can be used to further enhance spatial resolution and increase the signal difference between USPIO accumulating normal lymph nodes and metastatic lymph nodes retaining MR signal [18, 19].

MR imaging of the pelvis at 7-T is technically challenging due to severe signal voids that are caused by RF transmission interferences [20]. Time-interleaved acquisition of modes (TIAMO) [21] is a method that efficiently uses two different shim settings to acquire two complementary images that are combined into one image with an apparent homogeneous RF excitation. This method has been used in combination with water- and lipid-selective excitation to obtain high-resolution 7-T MR images of the pelvis in healthy volunteers [19]. Because imaging was performed with a low flip angle, T_2_^*^-sensitive gradient echo (GRE) sequence, this method can easily be adapted for USPIO-enhanced 7-T MR imaging without any issues with increased radiofrequency power deposition.

The aim of this paper is to demonstrate the technical feasibility of 7-T high-resolution USPIO-enhanced MRI of pelvic lymph nodes in patients with pelvic cancers.

## Methods

### Subjects

To illustrate the technical feasibility of our method, three patients with histologically proven prostate cancer were selected based on the presence of multiple small lymph nodes without USPIO uptake on their clinical 3-T examination. These patients were asked permission to perform a 7-T examination. Three additional patients with histologically proven rectal cancer were included before undergoing a total mesorectal excision and underwent 3-T as well as 7-T examinations (part of a larger patient trial of 20 patients, clinical trials identifier NCT02751606). The study was approved by the ethics review board (CMO region Arnhem-Nijmegen and the Ethics committee of the Medical Faculty of the University of Duisburg-Essen) and signed informed consent was obtained for inclusion into the study from all patients before participation. Patients were excluded when contra-indications for 7-T MRI (metal implants, epilepsy, abdominal circumference > 120 cm) or USPIO contrast (any prior contrast reaction, allergy to dextran or ferumoxtran-10, hemochromatosis, thalassemia, sickle cell anemia) were present and all inclusions were performed prospectively between June 2014 and October 2017. All patients (see Table 1 for clinical information) were scanned 24–36 h after an intravenous infusion of USPIOs (ferumoxtran-10, which is an investigational product produced by Radboudumc; from September 2015: SPL Medical B.V.) [9] of 2.6-mg iron per kg of bodyweight. For the infusion, the USPIOs were diluted in 100 cc saline solution and administered over 30 min using a Minisart NML 0.22-μm pore size filter (Minisart NML Syringe Filters 16,534-k; Sartorius AG). Patients were observed during and shortly after the 30-min contrast administration period for adverse reactions. On the day of the MRI scan, patients were asked whether any adverse reactions had occurred. All rectal cancer patients were scanned before surgical treatment. All patients received intramuscular smooth muscle and bowel relaxant and rectal cancer patients received a laxative (Table 2).

**Table 1:** Clinical patient data including the number and size of lymph nodes without USPIO signal decay per patient assessed on 7-T imaging. The staging data were obtained from the available clinical data

Patient	Sex	Age	Cancer	Stage	Therapy	Scan timing	Number of LNs without USPIO signal decay on 7-T	Mean short axis (range) of LNs without USPIO signal decay
1	Male	54	Prostate	pT3bN1Mx, Gleason 8	Prostatectomy + PLND	Before prostatectomy + PLND	48	2.7 mm (1.5–7)
2	Male	52	Prostate	pT3a R1 NxMx, Gleason 7, chemical recurrence	Prostatectomy, no PLND	After prostatectomy	24	3 mm (1.5–6)
3	Male	62	Prostate	cT1Nx, Gleason 3 + 3 (TRUS-guided biopsy)	No follow-up available	Before treatment	18	3.1 mm (1.5–7)
4	Male	77	Rectal	pT3N1Mx	TME (total mesorectal excision)	Before TME	9	3 mm (2–5)
5	Female	64	Rectal	pT1N1Mx	TME	Before TME	3	2.5 mm (2–3)
6	Female	55	Rectal	pT0N1M0, complete response of primary tumor to chemoradiation	Neoadjuvant chemoradiation followed by TME	Before TME, after chemoradiation	6	5.3 mm (3–8.5)

**Table 2:** Overview of the used MR pulse sequence parameters

	7-T				3-T	
	Rectal cancer patients		Prostate cancer patients		Rectal cancer patients	
	Water-selective imaging	Lipid-selective imaging	Water-selective imaging	Lipid-selective imaging	Water-selective imaging	T1W
Voxel size (mm^3^)	0.68 × 0.68 × 0.68	0.68 × 0.68 × 0.68	0.66 × 0.66 × 0.66	0.66 × 0.66 × 0.66	0.85 × 0.85 × 0.85	0.9 × 0.9 × 0.9
FOV (mm^3^)	260 × 260 × 174	260 × 260 × 174	210 × 210 × 169	210 × 210 × 169	328 × 328 × 190	350 × 350 × 173
Acquisition mode	3D	3D	3D	3D	3D	3D
Matrix	384 × 384 × 256	384 × 384 × 256	320 × 320 × 256	320 × 320 × 256	384 × 384 × 224	384 × 384 × 192
Acquired TEs (ms)	2.28, 4.76, 7.24, 9.72, 12.2	2.3	2.1, 4.19, 6.21, 8.3, 10.32	2.0	3.6, 7.0, 10.4, 13.8, 17.2	2.5
Reconstructed TEs (ms)	0, 8, 14	n.a.	0, 8, 14	n.a.	0, 8, 14	n.a.
TR (ms)	16	5.9 or 5.4	14	5.2	21	5.8
Bandwidth (Hz)	501	766	625	625	590	305
Acceleration***	3 × 2	3 × 1	2 × 2	2 × 2	3 × 1	2 × 1
Acquisition time (min)	9:30	8:08 or 7:26	8:24	2:50	11:28	4:56
Excitation	Water-selective	Lipid-selective with partial water excitation	Water-selective	Lipid-selective	Water- and slab-selective	Slab-selective
Flip angle	* TIAMO RSS FA ≈ 16°	* TIAMO RSS FA ≈ 21°	** n.a.	** n.a.	10°	10°
Bowel preparation	10-mg bisacodyl orally on the evening before scan and 20-mg butylscopolamine i.m. just before scan		20-mg butylscopolamine i.m. just before scan		Identical to 7-T rectum	

**Fig. 1:**
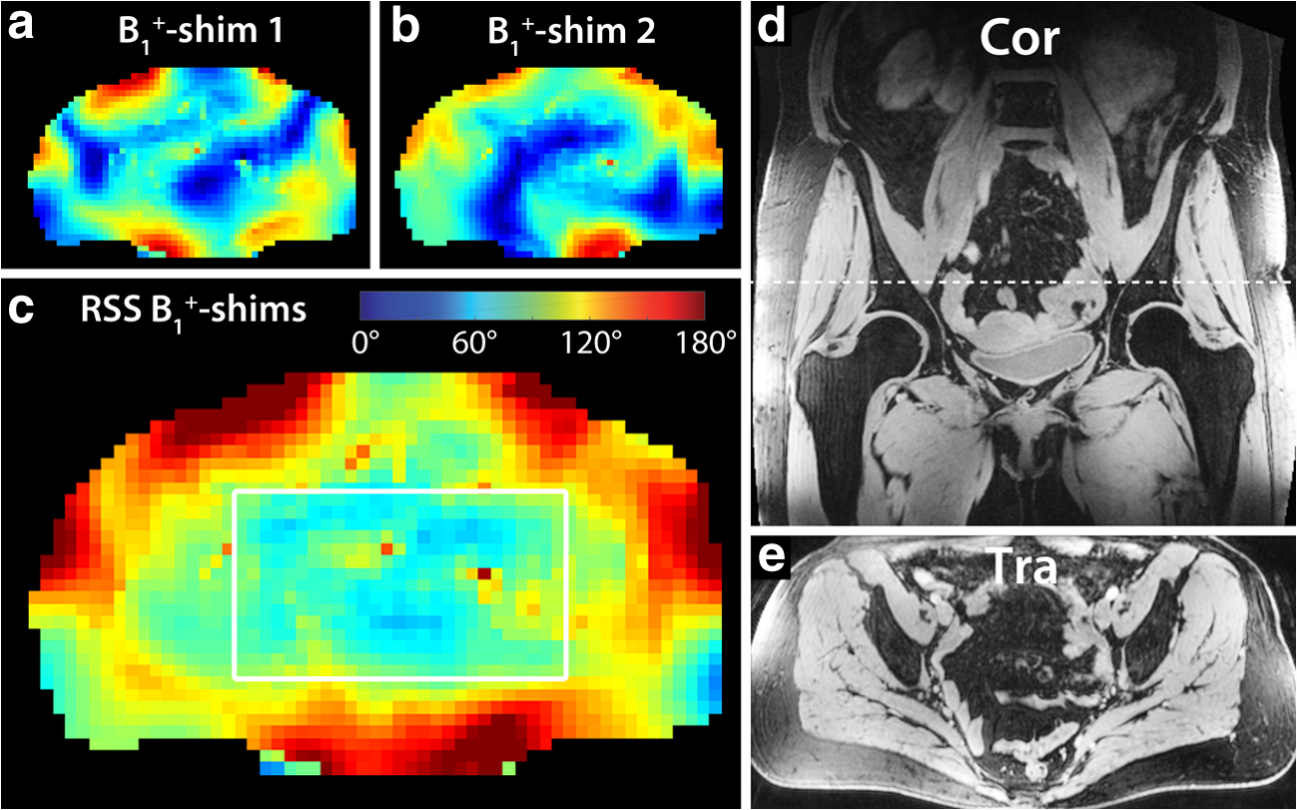
The TIAMO flip angle maps corresponding to the two individual B_1_^+^ shim settings (**a** and **b**) and the combined root sum of squares (RSS) flip angle map (**c**) in patient 5 (male, 64 years old, rectal cancer). The white box in **c** indicates the ROI in which the flip angle distribution was evaluated. The two complementary shim settings produced a RSS homogenous flip angle distribution over a large FOV (water-selective GRE overview image example in **d** and **e**) (see also Table 2). A single axial slice (**a**–**c**) was used for B_1_^+^ shimming (indicated by dashed line in coronal image **d**). The corresponding axial overview GRE image is depicted in **e**

*RSS flip angle over two TRs; based on the estimation as discussed in image analysis section and Fig. 1

**No B1TIAMO was performed in these measurements to determine the actual FA

***Generalized autocalibrating partial parallel acquisition (GRAPPA)

### Measurement setup

All 7-T MRI measurements were performed on a whole-body research MR system (MAGNETOM 7-T, Siemens Healthcare GmbH). The patients with prostate cancer were examined using a custom-built 8-channel transceiver (8Tx/8Rx) body-array coil [22] and the three patients with rectal cancer were scanned using a custom-built combined 8-channel transmit / 32-channel receive (8Tx/32Rx) body array [23], both with meander-type microstrip elements [24]. TIAMO shimming was performed in all subjects, using a phase and amplitude RF shimming algorithm to optimize two complementary B_1_^+^ modes for root-sum-of-squares (RSS) homogeneity [25]. For measurements with the 8Tx/8Rx coil, B_1_^+^-shimming was based on a series of single-slice low flip angle (FA) GRE images of the individual coil elements with the slice positioned transversally through the pelvis. The 8Tx/32Rx measurements were performed using the B1TIAMO B_1_^+^ mapping technique [26], which, next to relative B_1_^+^ maps, also yielded absolute B_1_^+^ results.

The 3-T MRI scan (MAGNETOM Prisma-Fit, Siemens Healthcare GmbH) in patients with rectal cancer was performed with table and body-phased array coils (Table 2).

### Imaging

After a localizer, B_0_ and B_1_^+^ shimming, a large field of view (FOV) 3D slab-selective GRE image was acquired for an anatomical overview (FOV 384 × 384 × 192 mm^3^, matrix 384 × 384 × 192, repetition time (TR) 4.1 ms, echo time (TE) 1.81 ms, RSS FA of 6° over 2 TRs, acquisition time 1:33 min). For water-selective imaging, five echoes were acquired (referred to as “original images”) using a multi-gradient echo (mGRE) sequence. A monoexponential decay model was fitted to the signal decay across the five echoes for every pixel in these images using a weighted linear least squares (WLLS) algorithm [19, 27], yielding maps of the R_2_^*^ relaxation rate (where R_2_^*^ = 1/T_2_^*^). The WLLS weights the measurement points based on their signal level to account for the non-zero mean of noise in magnitude images. Based on the fit results, computed echo time images (referred to as “computed TE images”) were reconstructed at TEs 0, 8, and 14 ms. In addition, a lipid-selective imaging series was performed for an additional complementary anatomical overview of the lower abdomen at the same spatial resolution. The FOV covered at least the area from the aortic bifurcation to the bottom of the urinary bladder (for prostate imaging) and rectum (for rectal imaging). The sequence parameters are summarized in Table 2.

Both 3-T and 7-T scans were performed within the 24–36-h timeframe after USPIO contrast administration, with no more than 5 h between the start of the studies.

### Image analysis

As RF transmit inhomogeneity leading to inhomogeneous FA distributions is a major issue in 7-T body imaging, FA maps were acquired with the B1TIAMO technique to evaluate the individual FA distributions. We calculated the effective FA distribution (mean ± standard deviation (SD)) in the water- and lipid-selective imaging by determining the FA range within a region of interest (ROI) relevant for lymph node imaging.

To evaluate the T_2_^*^ signal decay within lymph nodes, ROIs were drawn within the lymph nodes on the original TE = 2.1 ms images and copied to the original images at the other TEs. A WLLS fit of the voxel-averaged signal at different TEs within these ROIs was performed. To account for the Rician noise distribution of magnitude images, the average intensity of the noise of a nearby ROI within lipid tissue (no signal on the water-selective images) was subtracted from each lymph node ROI signal. The uncertainty of the R_2_^*^ value in each node was calculated based on the fitting accuracy and expressed as a standard deviation.

To provide initial descriptive findings of visible lymph nodes in the six patients, the images were evaluated by one of two radiologists (7 and 11 years of experience) to assess the USPIO signal decay of lymph nodes on the isotropic 3D datasets in at least two orientations. The lymph nodes were identified on a combined reading of lipid-selective images and computed TE = 0 ms images of water-selective scans. Nodes were annotated as “without USPIO signal decay” if they retained high signal intensity on the computed TE = 8 ms imaging according to a “metastatic” or “possibly metastatic” pattern as described in Heesakkers et al [5]. The short axis of lymph nodes without USPIO signal decay was evaluated on the computed TE = 8 ms image by the coordinating researcher (B.W.J. Philips).

## Results

No adverse drug reactions occurred during contrast administration and all patients accepted the 7-T MRI scan without any complaints. We performed susceptibility-sensitive water- and lipid-selective pelvic imaging at 7-T without suffering from any RF-related signal voids in any of the patients, using the TIAMO technique (Fig. 1). Total measurement time, including patient and RF coil positioning and calibration measurements, was less than 1 h. Only at the superior and inferior edges of the large FOV images, some reduced signal was observed due to the limited size of the RF coil array in this dimension. Within the depicted ROI (Fig. 1c), the calculated RSS FA over two TRs was 16.2 ± 2.8° (mean ± SD) for the water-selective imaging and 21.0 ± 3.6° for the lipid-selective imaging.

**Fig. 2:**
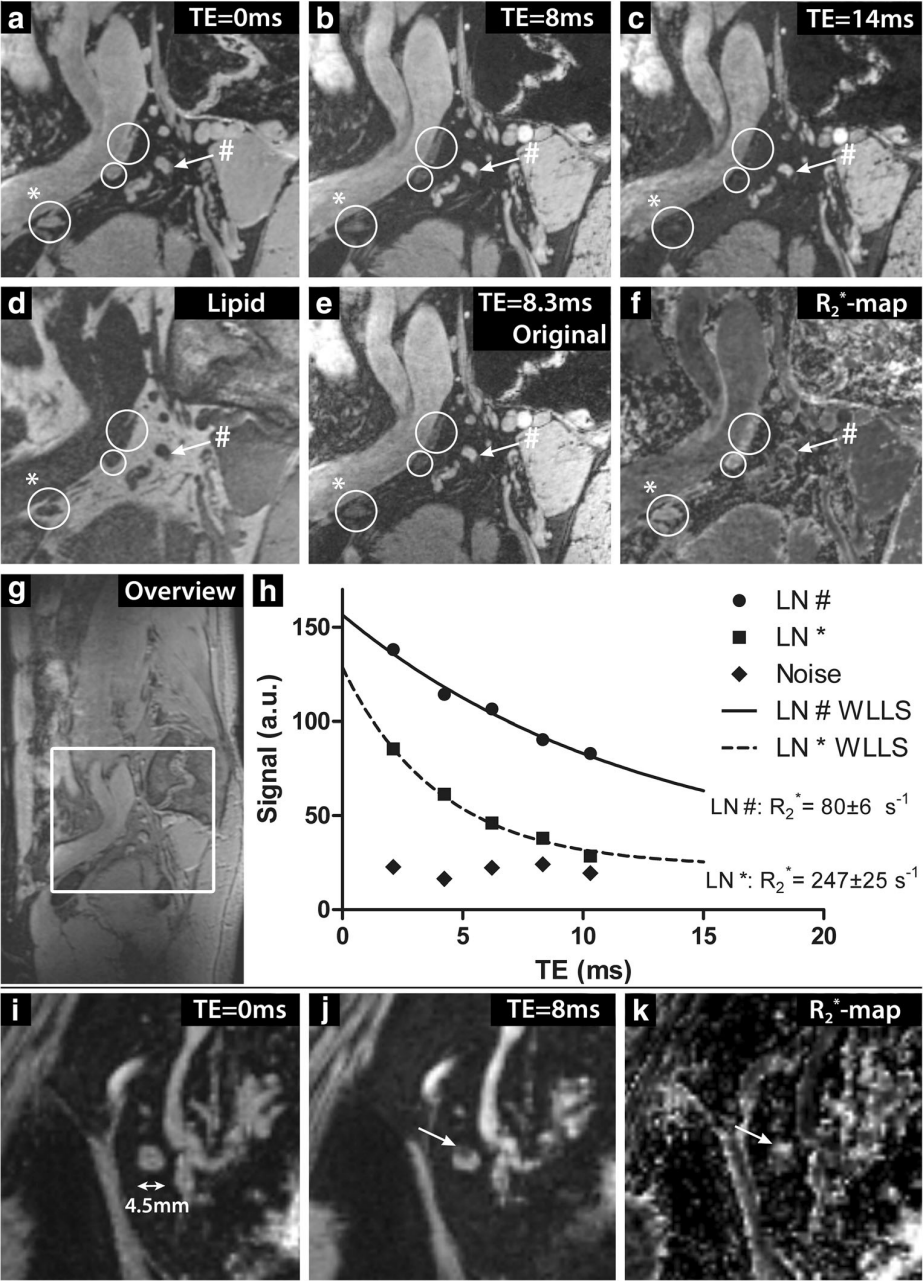
Imaging at 7-T of patient 1 (male, 54 years old, prostate cancer). A sagittal image is shown at different computed TEs (**a**–**c**), lipid-selective imaging (**d**) and the original mGRE water-selective imaging at TE 8.3 ms (**e**). In addition, a map of R_2_^*^ relaxation rates obtained from the WLLS fit is depicted (**f**). Three lymph nodes with USPIO signal decay (white circles) and one lymph node without USPIO signal decay (white arrow) were annotated. A sagittal overview image is also shown (**g**). The GRE signal decay of a normal LN with USPIO signal decay (annotated in images **a**–**f** with asterisk) and a suspicious LN with low USPIO signal decay (annotated with number sign) are depicted in **h**. The lymph node marked with a number sign showed a slow signal decay with a low R_2_^*^ value of 80 ± 6 s^−1^, whereas the lymph node marked with an asterisk showed a fast R_2_^*^ decay with a high R_2_^*^ value of 247 ± 25 s^−1^. In addition, a lymph node is shown with homogenous high signal intensity at the computed TE = 0 ms image (**i**) that shows partial USPIO signal decay in a small region, indicated by the arrow. In this region, signal intensity on the TE = 8 ms image (**j**) is low and the R_2_^*^ values on the R_2_^*^ map (**k**) are high, whereas the rest of the lymph node shows low USPIO signal decay indicated by high signal intensity at TE = 8 ms and low R_2_^*^ values

Reading the combination of lipid series and water-selective TE = 0 ms images, lymph nodes with high and low USPIO signal decay could be detected. A range of 3–48 lymph nodes without USPIO signal decay were found in the six patients with a maximum short-axis diameter within the range of 1.5–8.5 mm (Table 1). Normal lymph nodes, which show high USPIO uptake, appeared dark on the water-selective computed TE imaging at long TE due to the strong R_2_^*^ decay effectuated by the USPIOs. This resulted in high R_2_^*^ values on the R_2_^*^ maps. Suspicious lymph nodes, without USPIO signal decay, had a high signal intensity at long TE and low R_2_^*^ values, as they did not experience the strong R_2_^*^ effect of USPIOs. With extrapolation to TE = 0 ms, the signal intensity of lymph nodes with high USPIO uptake was recovered, such that computed TE imaging was also used for detecting normal lymph nodes (Fig. 2). Computed TE images had a higher SNR than the original images, but similar contrast (Fig. 2b, e). Lymph nodes that take up USPIO contrast only within a small part of the cortex could also be observed, as illustrated by the 4.5-mm lymph node shown in Fig. 2i–k, which shows distinct regions of high and low USPIO signal decay.

**Fig. 3:**
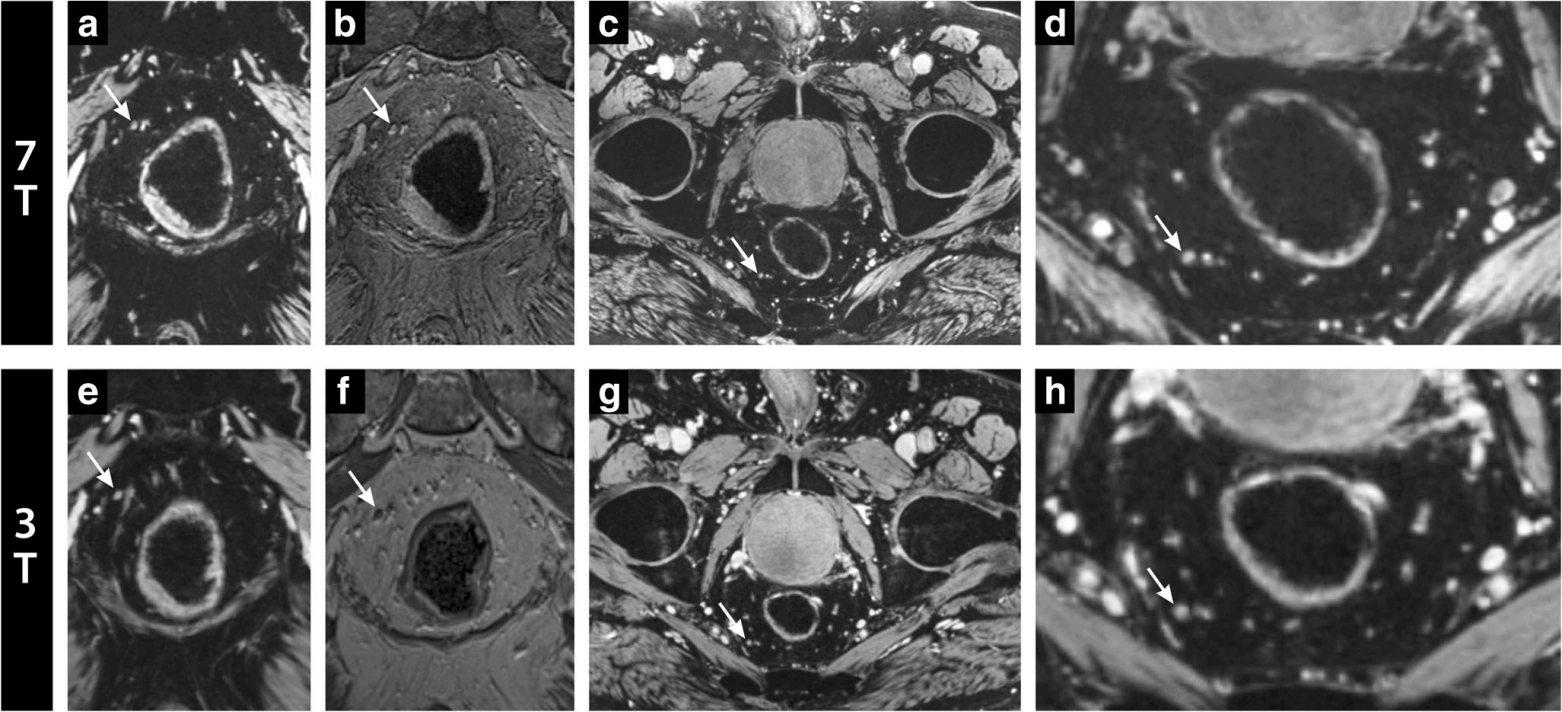
Water- (computed TE = 8 ms) (**a**, **c**–**e**, **g**, and **h**) and lipid-selective imaging (**b** and **f**) at 7-T (top) and 3-T (bottom) in coronal (**a**, **b**, **e**, and **f**) and axial direction (**c**, **d**, **g**, and **h**) of patient 4 (male, 77 years old, rectal cancer). A lymph node with low USPIO signal decay and bright signal intensity is annotated with a white arrow and was marked as “without USPIO signal decay.” Note particularly the better visibility and delineation of the pathological lymph node and other structures within the mesorectal area at 7-T

**Fig. 4:**
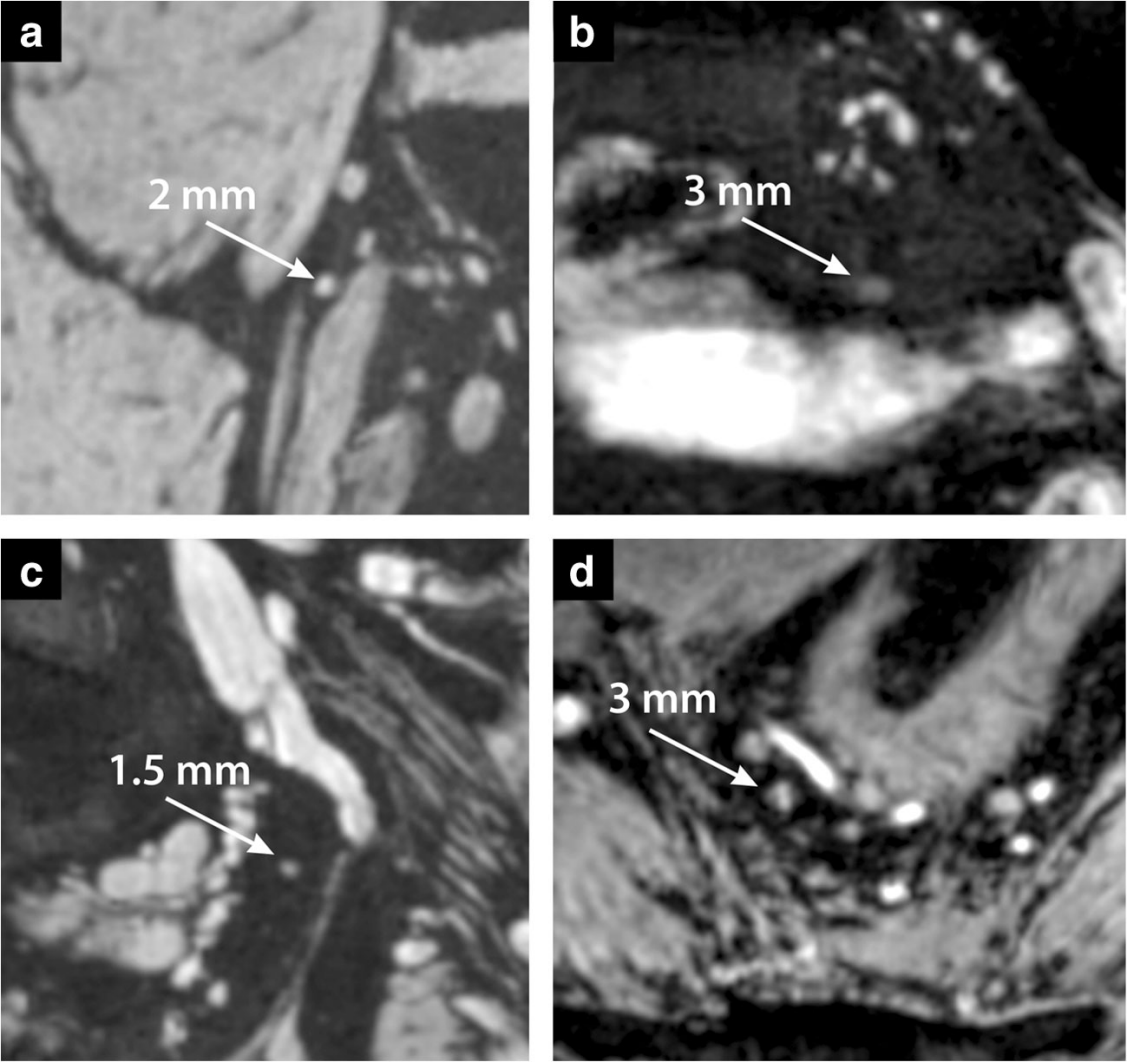
Coronal (**a**–**c**) and transversal (**d**) zoomed computed TE = 8 ms images at 7-T of the four patients not depicted in Figs. 2 and 3. **a** Patient 2 (male, 52 years old, prostate cancer). **b** Patient 3 (male, 62 years old, prostate cancer). **c** Patient 5 (female, 64 years old, rectal cancer). **d** Patient 6 (female, 55 years old, rectal cancer). The images indicate a lymph node and its short axis that was marked as lymph node without USPIO signal decay by a radiologist (white arrow). Note how these lymph nodes without USPIO signal decay, although small, are clearly visible due to bright signal and can be delineated well from other structures. The node identified in **d** shows signal heterogeneity, which could indicate partial USPIO signal decay within this node

Water-selective 7-T lymph node imaging was performed at a higher spatial resolution in a similar acquisition time compared with 3-T. In the three patients with rectal cancer examined on 3-T and 7-T, vessel and node delineation were sharper at 7-T (example in Fig. 3). The high spatial resolution at 7-T enabled visualization of lymph nodes down to sizes of 1.5 mm short axis (Fig. 4). In the water-selective images at 3-T, the blood vessels were somewhat hyperintense due to the T1-shortening effect of USPIO contrast in the blood pool.

## Discussion

This work demonstrates the technical feasibility of USPIO-enhanced pelvic MRI at 7-T and shows its potential for diagnosing small lymph node metastases in patients with rectal cancer and prostate cancer. Our approach mitigates many of the technical challenges associated with 7-T body imaging, enabling large FOV USPIO-enhanced imaging of the pelvis without any RF-related signal voids.

Water-selective mGRE imaging lends itself particularly well for ultrahigh field, as it exploits the T_2_^*^ effect of USPIOs and can be performed at low FAs to reduce specific absorption rate (SAR) issues. It can be performed reliably using the TIAMO technique and can already compete with our clinical protocol at 3-T. It provides a higher spatial resolution which may allow the assessment of USPIO uptake in normal-sized lymph nodes down to 1.5 mm in short-axis diameter or the evaluation of partial uptake within a lymph node.

The B1TIAMO B_1_^+^ mapping technique enables patient-specific flip angle calibration, such that a similar SNR and contrast are obtained in all patients. It shows relatively homogenous root-sum-of-squares FA distributions.

A general observation of GRE imaging at 7-T is the shorter overall T_2_^*^. This is compensated by a shorter T_2_^*^ of lymph nodes with USPIO uptake at 7-T and it may even lead to a contrast advantage at higher field strengths. Because this T_2_^*^ effect is field strength–dependent, the TE should be adjusted as well to provide optimal distinction between normal and suspicious lymph nodes at 7-T. Current literature is, however, inconclusive about the exact TE that should be used for optimal T_2_^*^-weighting, reporting values in the range of 11–27 ms at 1.5-T [5, 28–30]. Furthermore, these reported values often correspond to effective TEs, which are obtained by the sum of squares combination of several images with different TEs, resulting in ambiguous T_2_^*^ contrast. We therefore used computed TE imaging [19], in which a voxel-wise exponential fit is performed over the TEs, pooling information from all images without losing the T_2_^*^ contrast information. This approach allows calculation of images at arbitrary TE, so that an optimal computed TE for detecting lymph node metastases can be assessed retrospectively (in validation studies).

As the acquisition time of the current GRE imaging protocol is about 9 min, bowel motion can cause motion artifacts despite administration of butylscopolamine, rendering mesenteric lymph nodes difficult to assess. Fortunately, this did not appear for iliac or mesorectal lymph nodes, particularly when combined with butylscopolamine and bowel preparation [31]. Lymph nodes in prostate and rectal cancer were therefore well visible.

7-T MRI offers advantages in SNR and the susceptibility to USPIOs, which can be used to increase spatial resolution and sensitivity to USPIO uptake compared with current clinical 3-T MRI. Its main downside at this time is its limited availability and absence of a standard RF body coil configuration and standard body imaging methods.

This work shows preliminary observations of the use of USPIO-enhanced MRI at 7-T in a small number of patients. Although USPIO-enhanced MRI was validated for the detection of pelvic lymph nodes in previous studies [5], there is not much literature on its validity in the small lymph nodes that are visualized with high-resolution 3D MRI. The patients in this study, particularly those with prostate cancer, show a large number of small lymph nodes without USPIO signal decay. A reason for this large number of nodes could be our selection of prostate cancer patients with a high number of suspicious lymph nodes based on a clinical USPIO-enhanced MRI scan. Whether all these lymph nodes were indeed metastatic could not be validated in this work: no systematic follow-up or node-to-node pathological data was available. Although lymph nodes with USPIO signal decay were found in all patients, we cannot be sure that our method is sensitive enough to visualize small quantities of USPIO uptake robustly. Moreover, as is known for inguinal lymph nodes [32], there could be regional differences in lymph node USPIO uptake. Larger patient studies with standardized radiological evaluation are therefore necessary to demonstrate the clinical feasibility of USPIO-enhanced MRI at 7-T for detecting lymph node metastases, particularly in normal-sized lymph nodes. To perform a validation on lymph nodes of this size requires a very systematic and meticulous correlation between imaging and pathology in a relatively large group of patients and is therefore not within the scope of this technical note. Another possibility would be to compare the results of USPIO-enhanced 7-T MRI with other imaging methods such as PET-CT or diffusion-weighted imaging (DWI). These methods, however, lose sensitivity for smaller lymph node metastases [33] and whole-pelvis DWI at 7-T is technically very challenging.

In conclusion, 3D large field-of-view lymph node MR imaging at 7-T is technically feasible and permits assessing USPIO signal decay in normal-sized lymph nodes throughout the lower abdomen in a patient suffering from primary pelvic cancers. Its clinical effectiveness needs to be evaluated in further clinical studies.

## Abbreviations

B1TIAMO: B_1_^+^ mapping technique for TIAMO B_1_^+^ shim calculation
FA: Flip angle
FOV: Field of view
GRE: Gradient echo
PLND: Pelvic lymph node dissection
RF: Radiofrequency
ROI: Region of interest
RSS: Root sum of squares
RSS FA: Root sum of squares flip angle
SNR: Signal-to-noise ratio
TE: Echo time
TIAMO: Time interleaved acquisition of modes
TME: Total mesorectal excision
USPIO: Ultrasmall superparamagnetic particles of iron oxide
WLLS: Weighted linear least squares
